# A conceptual framework integrating mechanisms linking nut consumption and energy balance

**DOI:** 10.3389/fnut.2026.1834816

**Published:** 2026-07-02

**Authors:** Tim D. Cassettari, Carlene S. Starck, Emma L. Beckett, Flávia Fayet-Moore

**Affiliations:** 1 FOODiQ Global Australian Catholic University, Sydney, NSW, Australia; 2 School of Behavioural and Health Sciences, Australian Catholic University, North Sydney, NSW, Australia; 3 School of Environmental and Life Sciences, The University of Newcastle, Callaghan, NSW, Australia

**Keywords:** body weight, conceptual framework, energy balance, gut microbiota, insulin sensitivity, metabolizable energy, nuts, satiety

## Abstract

Although nuts are calorie-dense foods, meta-analyses of randomized trials show that nut consumption generally does not promote weight gain over the short term across commonly consumed nut types. In contrast, prospective cohort studies indicate that higher nut intake is associated with modest reductions in long-term risk of weight gain, although these findings do not establish causality. These findings are not easily explained by simplified interpretations of energy balance that primarily emphasize calorie content. This review examines the key mechanisms that may contribute to the relationship between nut consumption and body weight and proposes a conceptual framework that integrates these mechanisms within established energy balance models. Reduced metabolizable energy and increased energy compensation represent the most consistently supported mechanisms underlying the weight-neutrality of nut intake, with limited evidence suggesting potential modest increases in energy expenditure. Emerging evidence also points to potential metabolic adaptations, including modulation of gut microbiota and short-chain fatty acid production, improved insulin sensitivity, and lower inflammation, which may contribute to longer-term regulation of energy balance. Together, the framework illustrates how nuts may influence energy balance through pathways beyond their direct calorie contribution while remaining consistent with thermodynamic principles, emphasizing the importance of their broader nutritional composition and physical structure.

## 1 Introduction

Nuts (tree nuts and peanuts) defy simplified interpretations of energy balance: they are calorie-dense, providing approximately 550–720 kcal per 100 g (1), yet do not typically cause weight gain. This is supported by more than two decades of research, including systematic reviews of randomized controlled trials (2, 3) and prospective cohort studies that report a reduced risk of weight gain over time (3). Counterintuitively, dose–response analyses of clinical trials and prospective cohorts suggest that higher nut intake is associated with modest reductions in body weight (3). Proposed mechanisms include lower metabolizable energy derived from nuts than predicted by Atwater factors, and a reduction in energy intake from other foods across the day (4, 5). Beyond effects on body weight, higher nut intake lowers total and LDL-cholesterol (6) and is associated with a reduced risk of cardiovascular disease, cancer-related mortality, and all-cause mortality (7). In a network meta-analysis of randomized controlled trials comparing major food groups across ten intermediate cardiometabolic outcomes, including LDL-cholesterol, systolic blood pressure, and fasting glucose, nuts ranked highest based on cumulative benefit across outcomes (8).

The leading barriers to nut consumption include concerns about calorie-density and weight gain (9). In a survey of 710 members of the general New Zealand population, two-thirds agreed or strongly agreed that eating nuts “would cause me to gain weight” (10). Similar concerns existed among New Zealand health professionals (11). Such concerns are likely contributing to low levels of nut consumption globally. In Australia, mean intake is 4.6 g/day (11.75 g/day among nut consumers), with only 2% of people meeting the recommended daily target of 30 g or more (12). Similarly, low intakes have been reported in the United States (13, 14), New Zealand (15), Europe (16), and Latin America (17). A Global Burden of Disease analysis across 195 countries highlighted nuts as having the largest gap between current and optimal intakes of any food group (18).

These findings highlight a disconnect between evidence on nut consumption and concerns about calorie density and weight gain. Addressing this disconnect is important for aligning public and professional understanding with the evidence base. Common explanations have focused on the short-term effects of nuts on energy balance, including reduced metabolizable energy, energy compensation, and potential changes in energy expenditure (19–21). However, these explanations may not fully explain long-term effects on energy balance, and there is a range of additional mechanisms, including favorable shifts in gut microbiota, greater insulin sensitivity, and modest reductions in inflammation, which together may help to create a metabolic environment more conducive to stable energy balance over time.

In this review, we examine the key mechanisms that may contribute to the relationship between nut consumption and body weight and propose an integrative conceptual framework. A structured narrative synthesis was conducted to integrate evidence across heterogeneous mechanistic domains, study designs, outcomes, and timeframes. Mechanistic domains were identified through preliminary scoping of the literature, author expertise, and consideration of established energy balance pathways. Structured searches were developed for each domain and conducted in PubMed (MEDLINE) in May 2025, with no date limits applied. Searches were supplemented by reference list screening. Study selection prioritized higher levels of evidence, including systematic reviews and meta-analyses, followed by randomized controlled trials and mechanistic studies where appropriate. Where newer publications were narrower in scope, such as single-nut, population-specific, or non-systematic reviews, broader systematic reviews and meta-analyses were prioritized for general conclusions, with narrower evidence used as supportive context. Key findings were extracted and synthesized narratively to inform development of the conceptual framework. Search terms and selection considerations are provided in Supplementary Table S1.

## 2 Rationale for an integrated framework

The first law of thermodynamics states that energy cannot be created or destroyed, only transformed (22). Around the turn of the 20th century, Atwater and Rosa quantified human energy balance through landmark respiration calorimetry experiments that empirically demonstrated this law (23). Around this time, Atwater also established the metabolizable energy values for protein, fat, and carbohydrate, which still underpin modern energy labeling (24). In the mid-20th century, simple linear frameworks were developed to predict body weight trajectories from changes in energy intake and expenditure. A notable example was Wishnofsky’s paper, which estimated that 1 pound (~0.45 kg) of body fat contained roughly 3,500 kcal (25). This estimate became widely adopted as the “3,500 kcal rule” and influenced historical approaches to weight-loss guidance (26). However, it has since been challenged by contemporary models of energy balance, which account for dynamic physiological adaptations (26, 27). Dietary guidelines of the 1980s and 1990s emphasized limiting high-fat, calorie-dense foods to reduce total caloric intake and weight-related health risks (28–31). This linear and static view of energy balance remained influential even as evidence emerged that some high-fat and calorie-dense foods, such as nuts, extra-virgin olive oil, and avocados, confer health benefits (32–34).

Energy balance abides by thermodynamic principles but is increasingly understood as a dynamic system shaped by feedback loops between intake and expenditure, including adjustments in appetite and metabolic rate (35). Further, shifts in the metabolic status of an individual, encompassing factors such as insulin sensitivity, brown adipose tissue activity, the gut microbiome composition, level of chronic inflammation, and shifts in the circadian rhythm, can influence energy balance through indirect metabolic pathways (36–39). These shifts imply that two individuals of the same body weight and consuming isoenergetic diets may experience different weight-related outcomes, further reflecting the complexity of human metabolism, rather than a violation of thermodynamic principles. In addition, associations between specific foods and long-term weight gain are not well predicted by simplified energy balance-based metrics such as total fat or calorie density (40, 41), suggesting that the effects of foods on energy balance operate beyond their caloric value alone. Dietary patterns promoted as supportive for health, including the Mediterranean (42, 43) and EAT-Lancet (44, 45), explicitly encourage calorie-dense foods with established health benefits, with EAT-Lancet including nuts and unsaturated oils at quantities up to 75 g and 80 g/day, respectively (45).

**Figure 1:**
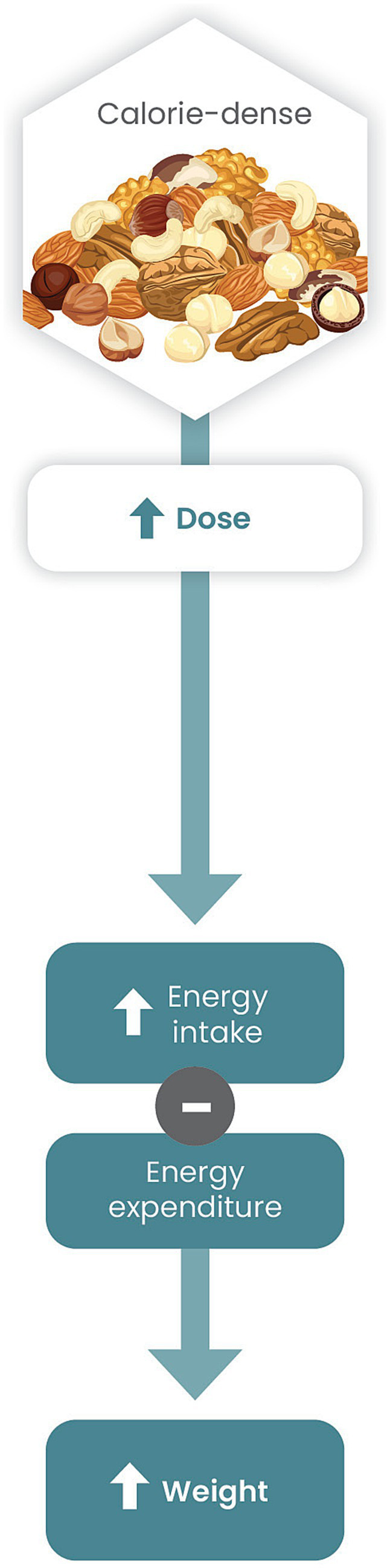
Simplified representation of energy balance as commonly conveyed in public-facing dietary messaging and label-based frameworks. This simplified schematic states that because nuts are calorie-dense, their consumption inherently increases total energy intake, resulting in positive energy balance and weight gain. In this framework, greater doses of nuts lead to a greater total energy intake and thus weight gain. Nutritional and structural components of nuts, and their potential to influence energy balance beyond their direct caloric contribution, are not considered. While this simplified representation is consistent with thermodynamic principles, it does not capture the dynamic, adaptive, and multifaceted process described in contemporary energy balance models.

Despite evidence that nuts confer health benefits without increasing body weight, concerns about their calorie density persist among health professionals and the public. In practice, public-facing dietary messaging commonly positions 30 g/day as a reference amount that can be interpreted as an upper limit, with recommendations often accompanied by caution around calorie content (46–48). Within national dietary guidelines, linear energy modeling and calorie-focused language may unintentionally reinforce caution and smaller serve sizes for nuts due to their calorie density, even where they are acknowledged as a healthful, nutrient-rich food. Front-of-pack labeling schemes that include calories or kilojoules as part of their algorithms (e.g., Health Star Rating in Australia/New Zealand, Nutri-Score in Europe, and warning labels in Latin America) may penalize some nuts and nut products, particularly where energy density is weighted without sufficient consideration of overall nutritional profile. This may further contribute to the discouragement of nuts in both reformulation and intake. Such calorie-focused messaging for nuts is built on the simplified interpretation that calorie-dense foods inherently increase energy intake, leading to positive energy balance and weight gain (Figure 1). An integrated framework is therefore needed to explain how nuts can influence energy balance through mechanisms beyond calorie content alone.

## 3 The evidence on nuts and body weight

Evidence from at least 19 systematic reviews consistently demonstrates that nut consumption does not lead to weight gain. This includes evidence from controlled trials on total nuts (2, 3, 49–53), walnuts (54–56), almonds (57–59), pistachios (60, 61), Brazil nuts (62), cashews (63), and peanuts (64), which consistently report no increases in weight gain, as well as observational studies indicating protective associations for total nut intake (3, 65). This pattern has been observed across specific population and intervention contexts, including trials in adults with type 2 diabetes (50) or atherosclerotic cardiovascular disease (49), trials with and without dietary substitution instructions (2), and trials conducted in the context of energy restriction (53). The most comprehensive of these reviews, published by Nishi et al. (3), assessed total nut intake across 86 randomized controlled trials (RCTs) and six prospective cohort studies. Certainty of the evidence was assessed using the GRADE (Grading of Recommendations Assessment, Development, and Evaluation) approach. Across included RCTs, where nut intake spanned 5–100 g/day and had a median duration of 8 weeks (range 3–104 weeks), there was a high degree of certainty that nut consumption had no effect on body weight (mean difference [MD] 0.09 kg; 95% CI −0.09 to 0.27 kg), with findings robust in sensitivity analyses. Effects were consistent for body mass index (BMI) and body fat (high certainty), and waist circumference, waist-to-hip ratio, and visceral adipose tissue (moderate certainty). Subgroup analyses showed consistent results across comparator types and between roasted versus raw and salted versus unsalted nuts. The available evidence is unevenly distributed across nut types, with the majority (>50%) of trials focusing on walnuts or almonds. Although subgroup analyses showed differences by nut type, these were small and may reflect between-study heterogeneity rather than true differences in effect. A 2021 network meta-analysis similarly reported no significant effect of individual nut types on body weight, BMI, or body fat, although almond interventions were associated with a small reduction in waist circumference compared with control (standardized mean difference −0.15; 95% CI −0.29 to −0.02) (51).

While causal evidence from RCTs indicates that nut consumption is weight-neutral, longer-term observational studies suggest modest associations between higher nut intake and reduced risk of overweight and obesity. One systematic review qualitatively reported inverse associations between long-term nut consumption and weight gain or risk of overweight/obesity (65). In the Nishi et al. review (3), which provided quantitative estimates, higher nut intake was associated with a 7% lower incidence of overweight/obesity (relative risk [RR] 0.93; 95% CI 0.88 to 0.98; moderate certainty), reduced risk of weight gain (≥ 5 kg) incidence (RR 0.95; 95% CI 0.94 to 0.96; moderate certainty), and lower incidence of elevated waist circumference (RR 0.72; 95% CI 0.65 to 0.80; moderate certainty). Median follow-up was 18 years (range 2.3 to 24 years). In a broader umbrella review comparing food groups, which drew on Nishi et al. for the nut evidence, nuts were identified alongside whole grains, legumes, and fruits as one of the few food groups associated with a reduced risk of overweight and obesity (40). However, these findings were based on fewer studies (n = 6) and should be interpreted cautiously. Nut consumers differ systematically from non-consumers in ways that may not be fully adjusted for, including higher physical activity levels, higher education and socioeconomic status, and healthier overall dietary patterns (66–68). Reverse causality is also possible if individuals at higher risk of weight gain limit nut intake because of their calorie density. Measurement error may also occur because self-reported dietary assessment methods can underestimate or misclassify intake (69).

Dose–response analyses from Nishi et al. further challenge simplified calorie-centric assumptions, although these estimates should be interpreted in the context of a single review (3). In RCT meta-regressions, higher nut intakes were associated with lower mean body weight (kg) (Greek beta: β −0.012, 95% CI −0.024 to −0.001, *p* = 0.04) and reduced body fat (%) (Greek beta: β −0.035, 95% CI −0.058 to −0.013, *p* < 0.01). These findings translate to approximately 360 g lower body weight and 1.05% lower body fat per additional 30 g of nuts consumed per day. No dose–response effects were observed in RCTs for BMI, waist circumference, waist-to-hip ratio, or visceral adipose tissue. In prospective cohort studies, higher nut intake was linearly and inversely associated with lower incidence of overweight/obesity, weight gain ≥ 5 kg, and elevated waist circumference. Together, the RCT and cohort findings suggest weight neutrality overall with small inverse associations for some weight-related outcomes. Differences between study designs may reflect potential time-dependent mechanisms, with cohort studies assessing longer-term outcomes, but do not establish causality and warrant cautious interpretation.

Understanding these findings requires consideration of the multiple mechanisms through which nuts may influence energy balance. In this review, mechanisms are grouped as direct modifiers, which alter energy absorption, intake, or expenditure, and indirect modifiers, which may influence the underlying metabolic environment over longer timeframes. These mechanisms are reviewed in the following sections and summarized in Table 1, which presents qualitative evidence classifications, quantitative estimates where available, and key limitations. Evidence classification reflects study design, quantity of evidence, consistency in direction, and directness to the proposed proximal mechanism, and should be distinguished from evidence that the mechanism causally translates into measurable changes in body weight, particularly for indirect modifiers of energy balance.

**Table 1:** Summary of evidence supporting each mechanism linking nut intake with energy balance within the conceptual framework.

Mechanisms	Proposed pathway and relevance to energy balance	Strength and consistency of evidence^1^	Magnitude/dose–response	Key active components	Key limitations	Key references
Metabolizable energy	Direct energy-balance pathway: lower metabolizable energy due to incomplete lipid digestibility; acts acutely with each exposure	Consistent; across 13 human and 11 *in vitro* studies but heterogeneous	5–26% lower ME than predicted by Atwater factors; absolute losses may increase as dose increases	Nut matrix / structure	Limited data for some nut types, inter-individual variability	Nikodijevic et al. (2023) (5)
Satiety and energy compensation	Direct energy-balance pathway: nuts modestly reduce hunger and offset subsequent energy intake; acute effects may contribute to weight stability over time	Consistent; across multiple SLRs and RCTs but heterogeneous	Mean compensation ~75%; variable (range −280 to +176%); dose-dependent reduction in one trial	Protein, fiber, texture, mastication effort	Self-reported intake; high inter-individual variability; contextual factors	Akhlaghi et al. (2020) (82), Nikodijevic et al. (2023) (4), and Baer et al. (2023) (19)
Energy expenditure and metabolism	Potential direct energy-balance pathway: nuts modestly increase DIT and fat oxidation post-consumption; possible effects on total EE over time remain uncertain	Limited; mixed findings across RCTs including 1 MA	Small, inconsistent effects; dose–response not established	Protein, unsaturated fatty acids	Few trials, heterogeneous methods, small magnitude of effect	Nikodijevic et al. (2023) (4), Franco Estrada et al. (2022) (95), and Mattes (2008) (21)
Gut microbiota and SCFA production	Indirect energy-balance pathway: nuts modestly alter gut microbiota and increase SCFA, potentially improving metabolic health and energy balance	Limited; emerging evidence across 3 SLRs (up to 28 trials)	Selective genus-level changes; no dose-response examined	Fiber, n-3 fatty acids, polyphenols, nut matrix	High variability; unclear effects at habitual intakes	Snelson et al. (2025) (90), Creedon et al. 2020 (102), and Fitzgerald et al. (2021) (103)
Insulin sensitivity and glycemic control	Indirect energy-balance pathway: nuts enhance insulin sensitivity and reduce fasting insulin; may influence energy partitioning and storage over time	Consistent; supported by SLR and MA of 40 RCTs	Modest improvements in HOMA-IR and fasting insulin; median dose 56 g/day	Unsaturated fatty acids, bioactives, fiber, protein, minerals	Null findings for glycemia, potential differences by nut type and health status	Tindall et al. (2019) (118) and Kim et al. (2017) (111)
Inflammatory markers	Indirect energy-balance pathway: nuts may modestly reduce ICAM-1, CRP, IL-6 and TNF-*α*; reduced inflammation may influence metabolic regulation and energy balance	Limited; suggestive evidence across 2 SLRs and MA (up to 23 RCTs)	Small or non-significant effects, greater reductions at ≥50 g/day and ≥12 weeks	Unsaturated fatty acids, bioactives; fiber, minerals	Heterogeneity in study design, comparators, and nut type	Rajaram et al. (2023) (137), Xiao et al. (2018) (134), and Neale et al. (2017) (133)

^1^Evidence strength and consistency refer to evidence that nut consumption influences the proximal mechanisms listed. Evidence classification is qualitative and reflects study design, quantity of evidence, and consistency of direction, and was not based on a formal certainty assessment. BMI, body mass index; CRP, C-reactive protein; DIT, diet-induced thermogenesis; EE, energy expenditure; g, grams; HOMA-IR, homeostatic model assessment of insulin resistance; ICAM-1, intercellular adhesion molecule-1; IL-6, interleukin-6; MA, meta-analysis; ME, metabolizable energy; SCFA, short-chain fatty acids; SLR, systematic literature review; TNF-α, tumor necrosis factor-alpha.

## 4 Direct modifiers of energy balance

Direct modifiers of energy balance include mechanisms through which changes lead to measurable effects on energy absorption, intake, or expenditure. These can occur acutely, within 24 h of consumption.

### 4.1 Reduced metabolizable energy

Metabolizable energy (ME) refers to the amount of energy available for use by the body after accounting for losses in stool and urine (24). The ME of a food is typically estimated using the Atwater factors, calculated by summing the energy contribution of each macronutrient multiplied by its corresponding energy factor. Limitations of the Atwater factors are well documented and include the small number of study participants, short study durations, and the use of experimental conditions and foods that do not reflect the general population or contemporary dietary patterns (24).

Increasing evidence indicates that Atwater-based calculations overestimate the ME content of nuts, partly due to incomplete lipid release from the nut matrix and reduced energy absorption during digestion. In a 2012 trial, the measured ME content of almonds was substantially lower than that predicted by Atwater factors (4.6 ± 0.8 kcal/gram vs. 6.0–6.1 kcal/gram, respectively) (70). This finding is supported by a 2023 SLR of 13 human and nine *in vitro* mechanistic studies (5). In the human studies, the ME of nuts was reported to be 5–26% lower than Atwater-predicted values. Reported reductions included almonds (up to 26%) (70, 71), walnuts (22%) (72), cashews (14%) (73), and pistachios (approximately 5%) (74). However, estimates for nut types other than almonds were based on fewer studies, and the precision of these estimates is therefore limited. In dose-comparison studies, higher nut doses were associated with similar or greater energy losses (70, 74, 75), suggesting that absolute energy loss may increase with dose.

*In vitro* studies suggest that this reduction in ME is largely explained by incomplete lipid release (5). Nuts have a distinctive food matrix, where lipids and protein are encapsulated within rigid, fibrous cell walls, resulting in incomplete digestion (76). More processed forms, such as roasted nuts, nut flours, or nut butters, and increasing the number of chews (77), exhibit greater cellular disruption and show higher lipid release (5, 78). In an *in vitro* digestion model, the extent of processing was a stronger predictor of lipid bioaccessibility than the type of nut (79).

Overall, the evidence indicates that the ME derived from nuts is lower than predicted by Atwater factors, particularly for whole nuts. This appears partly explained by the nut matrix, which limits lipid bioaccessibility and may contribute to the weight-neutral effects observed in randomized controlled trials.

### 4.2 Satiety and energy compensation

Energy compensation refers to the adjustment of energy intake after consumption of a given food (80). Early work by Alper and Mattes found that when participants added approximately 90 g of peanuts to their habitual diets, two-thirds of the energy supplied by the peanuts was offset (66% energy compensation) (81). This initial finding has been supported by systematic reviews (4, 82). A 2020 systematic literature review (SLR) and meta-analysis of 23 RCTs estimated mean energy compensation at approximately 75% (82), although heterogeneity across studies was high (*I*^2^ = 82.1%) and the pooled estimate should therefore be interpreted cautiously. A subsequent 2022 SLR of 16 acute RCTs (<24 h) reported similar results in qualitative analysis. Most studies had energy compensation between 0 and 100%, but there was a wide range across studies, from −280.5 to 176.4% (4). Heterogeneity may reflect a range of individual and contextual factors. Across studies, compensation was generally greater when nuts were consumed as snacks (4, 83) and among individuals with lower BMI (82). Effects may also vary by nut dose. In one study, almonds provided as a mid-morning snack of 0, 28, or 42 g produced dose-dependent decreases in energy intake at subsequent meals (84).

Satiety is the most likely explanation for these effects on energy compensation. In a 2020 meta-analysis, nuts modestly suppressed hunger ratings (−6.54 mm on a Visual Analogue Scale), with effects on fullness not significant (82). In one study, reductions in self-reported hunger were greater for nuts than for other foods matched by weight or volume (85). Individuals with lower baseline satiety responsiveness may experience more pronounced appetite responses to nuts (19). These effects are likely multifactorial, reflecting the protein, unsaturated fat, and fiber content of nuts, each of which has been linked to enhanced satiety effects (86–89). These properties may delay gastric emptying and influence appetite-related hormones, including cholecystokinin, glucagon-like peptide-1, glucose-dependent insulinotropic polypeptide, and ghrelin, although nut-specific evidence for direct hormonal effects remains limited (57). Fermentation of nut fiber and polyphenols may also increase short-chain fatty acid production (90), which may influence satiety through interactions with enteroendocrine cells and gut-brain signaling pathways (91). The structural properties of nuts, including their hard texture and intact cellular structure, may contribute to appetite regulation (19). In one study, increasing almond chews from 25 to 40 before swallowing enhanced hunger suppression (77), with mastication potentially influencing satiety through reduced eating rate and altered digestion kinetics (92).

Overall, the evidence suggests that nuts can reduce hunger and partially displace energy intake from other foods, often offsetting a substantial proportion of the energy they provide. However, the magnitude of compensation varies widely across studies and appears highly context dependent. Most trials are acute (<24 h), while longer-term estimates rely largely on self-reported dietary intake methods, including food records, which are prone to underestimation (93).

### 4.3 Energy metabolism and expenditure

Energy expenditure encompasses resting metabolic rate, physical activity (including both exercise and non-exercise activity), and diet-induced thermogenesis (DIT) (94). A 2022 SLR and meta-analysis (4) comprising five acute (< 24 h) RCTs assessed the impact of nuts on postprandial energy expenditure or DIT, with inconsistent findings. Two studies observed significant increases in postprandial energy expenditure compared with control, two found no difference, and one reported a significant reduction. A second SLR on acute energy metabolism and expenditure also reported mixed results (95). Across four acute studies, three reported inconsistent effects on postprandial energy expenditure and DIT, while in the fourth study, 25–35 g of walnuts increased fat oxidation and reduced carbohydrate oxidation after 8 h (96). Both nut type and the choice of comparator may influence outcomes. For example, peanuts with a high-oleic acid content increased postprandial energy expenditure relative to regular peanuts but not compared to biscuits (97).

Longer-term RCTs (2–12 weeks) have also examined effects on resting energy expenditure. In a pooled meta-analysis of five eligible studies (4), no significant effect was detected (weighted mean difference [WMD]: 28.6 kcal/day, 95% CI −10.7 to 67.8 kcal/day), although the pooled effect became significant when one heavily weighted study was removed. This study compared energy-restricted diets containing either 56 g/day peanuts or a control diet matched for energy intake without peanut supplementation (98). Differences in nut type and comparator choice may again partly explain inconsistencies across studies. Dose–response relationships were not explored.

Effects may also differ based on the dietary instructions provided. In an eight-week RCT in adults at higher risk for cardiovascular disease, adding pecans to a free-living diet increased postprandial DIT over 3.5 h within the addition group, while replacing isocaloric foods with pecans increased resting metabolic rate within the substitution group (99). The substitution arm also showed higher fat oxidation and a lower respiratory exchange ratio, indicating greater reliance on lipid metabolism; however, whether these acute or short-term changes translate into sustained changes in total energy expenditure has not been established.

Overall, current evidence suggests that nuts may have small effects on energy metabolism and expenditure, although findings are inconsistent, based on a limited number of studies, and limited to short-duration trials (≤ 12 weeks). Where increases in energy expenditure are observed, they may be attributable to the thermogenic properties of nut protein and the higher oxidative potential of unsaturated fatty acids (4, 95).

## 5 Indirect modifiers of energy balance

Indirect modifiers are mechanisms through which changes may influence energy balance by altering the underlying metabolic environment. These include adaptations in insulin sensitivity, inflammation, and the gut microbiota, that can in turn affect appetite regulation and how efficiently dietary energy is processed or stored over time. The mechanisms described in this section are supported by emerging and indirect evidence and are presented as potential contributors to longer-term energy balance. While biologically plausible, these pathways do not establish causal effects on body weight and should be interpreted cautiously.

### 5.1 Gut microbiota and short-chain fatty acid production

The gut microbiota and short-chain fatty acids are increasingly recognized as regulators of body weight. In a landmark 2013 study, germ-free mice that received fecal microbiota transplants from human twins discordant for obesity developed divergent body weight trajectories despite being fed identical diets (39). Evidence suggests that gut microbiota can influence host energy balance both through modulation of energy harvest and via systemic effects on metabolism, appetite, circadian rhythm, inflammation, gene regulation, and hormonal and immune pathways (100, 101).

Emerging evidence suggests that nut-induced changes in the gut microbiota may influence metabolic processes involved in body weight regulation. Three systematic reviews (90, 102, 103) of up to 28 clinical trials, including one with a meta-analysis of RCTs (102), have examined the effects of nut consumption on gut microbial composition. Intervention durations ranged from <1 week to 6 months and nut dose ranged from 28 to 100 g/day of whole nuts. Selective and modest shifts at the genus level, including increases in *Clostridium* and *Roseburia*, and decreases in *Parabacteroides*, were reported.

These microbiota changes have been associated with metabolic processes relevant to body weight regulation; however, causal pathways linking nut-induced microbiota changes to energy balance outcomes in humans remain uncertain. Clinical studies also report increases in butyrate and propionate following higher nut intakes (90, 104). These short-chain fatty acids may influence metabolism through appetite-related gut hormones and gut-brain neural circuits, glucose regulation and insulin sensitivity, lipid oxidation and energy expenditure, gut barrier function, and reduced chronic low-grade inflammation (105–107). Animal studies provide supportive mechanistic evidence, with supplementation of selected butyrate-producing bacterial species, including members of the *Clostridium* and *Roseburia* genera, shown to attenuate weight gain in some models (108–110).

These effects are plausible given that nuts provide fiber, polyphenols, and unsaturated fatty acids including monounsaturated and n-3 polyunsaturated fatty acids, that may influence gut microbiota composition and metabolic activity (103, 111, 112). The intact nut structure may also contribute. This is supported by one study reporting that whole or chopped almonds had more pronounced effects on the gut microbiota than almond butter (113). However, responses may vary by baseline microbiome composition, habitual dietary patterns, and broader dietary context (114), and whether nut-induced microbiota or SCFA changes contribute meaningfully to long-term energy balance remains uncertain.

### 5.2 Insulin sensitivity and glycemic control

Insulin sensitivity reflects the efficiency with which target tissues respond to insulin stimulation (115). Impaired insulin sensitivity (insulin resistance) can promote hyperinsulinemia and is commonly associated with higher body weight (116, 117). Insulin sensitivity has been proposed as a key metabolic regulator of energy balance, influencing nutrient partitioning, fat storage, and appetite signaling (116, 117).

A large 2019 SLR and meta-analysis of 40 RCTs in adults with and without type 2 diabetes reported that nut consumption significantly improved markers of insulin sensitivity (118). Across 19 trials, there were modest decreases in the homeostatic model assessment of insulin resistance (HOMA-IR; WMD −0.23, 95% CI −0.4 to −0.06). Fasting insulin was also reduced (WMD −0.40 μIU/mL, 95% CI −0.73 to −0.07 μIU/mL), while effects on glycated hemoglobin (HbA1C) and fasting glucose were non-significant. Results for HOMA-IR were robust in sensitivity analyses and did not differ by nut type, dose, or change in participant body weight. Median nut doses were higher than usual intakes (52 g/day; range 20–128 g/day). Interventions ranged from 4 weeks to 12 months (median 3 months). Smaller reviews on individual nut types (63, 119, 120) and specific populations (49, 59) have produced mixed or null results, likely reflecting smaller study numbers or differences by participant health status.

Improvements in insulin sensitivity and reductions in fasting insulin may relate to the unsaturated fatty acids (111, 118), polyphenols including proanthocyanidins and ellagitannins (121, 122), protein (123), fiber (124), and micronutrients such as selenium, magnesium and zinc in selected nuts (125). Microbiota-derived short-chain fatty acids may also provide an additional pathway linking nut intake with insulin sensitivity (126). Overall, nuts appear to modestly enhance insulin sensitivity and reduce hyperinsulinemia, but the clinical significance of these effects in relation to body weight remains uncertain.

### 5.3 Inflammatory markers

Higher body weight is associated with elevated inflammatory markers (127). Emerging evidence suggests a bidirectional relationship, in which weight gain can promote chronic low-grade inflammation, and chronic inflammation may in turn contribute to metabolic dysfunction, impaired insulin sensitivity, and altered energy balance (128–131). In a transgenic rat model, chronic elevation of human C-reactive protein (CRP) promoted adult-onset obesity and was accompanied by changes in energy expenditure, thyroid hormones, gut microbiota, and immune responses (132).

Two systematic reviews have examined the effects of total nuts on inflammatory markers in healthy populations (133, 134). One was a 2018 meta-analysis of 23 RCTs (134), spanning 4 to 48 weeks, which reported modest reductions in intercellular adhesion molecule-1 (ICAM-1) (14 trials, WMD −0.17 ng/mL, 95% CI −0.32 to −0.03 ng/mL). Results were robust in sensitivity analyses. Non-significant reductions were reported for other inflammatory markers including vascular intercellular adhesion molecule-1 (VCAM-1), interleukin-6 (IL-6), CRP, and tumor necrosis factor-alpha (TNF-*α*). The other was a 2017 SLR which reported similar results, although reductions in ICAM-1 were non-significant (133).

The lack of consistent significant reductions in inflammatory markers may relate to dose, duration, or comparator choice. In sub-analyses, significant reductions for CRP were seen in higher doses (≥50 g/day) (133, 134). Similarly, for ICAM-1 and VCAM-1, reductions were seen in studies of longer durations (≥ 12 weeks). Some trials used comparators with anti-inflammatory properties (e.g., olive oil), which may have masked potential effects. Effects may also be limited to select nut types. More recent nut-type specific systematic reviews have reported reductions in selected inflammatory markers, including CRP and IL-6 for walnuts (135), and IL-6 and TNF-*α* for almonds (136). Potential anti-inflammatory effects may relate to unsaturated fatty acids, polyphenols, dietary fiber, and minerals, including selenium and copper (137).

## 6 Redefining nuts and energy balance: the conceptual framework and its limitations

**Figure 2:**
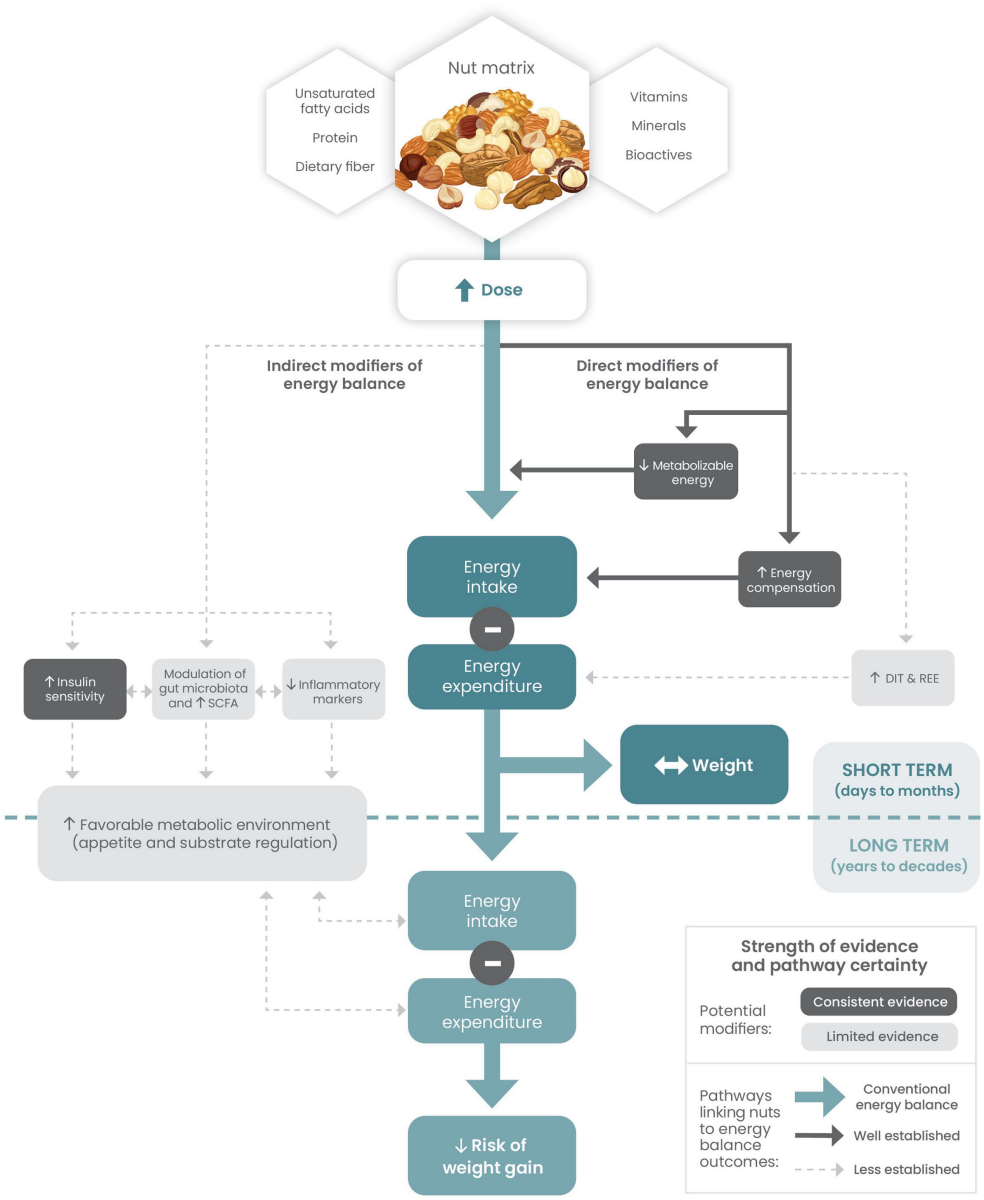
Conceptual framework illustrating proposed mechanisms through which nut consumption may influence energy balance. The framework integrates direct and indirect mechanisms proposed to help explain the typically weight-neutral effects of nuts, while remaining consistent with thermodynamic principles and established dynamic energy balance models. Direct mechanisms include energy compensation, reduced metabolizable energy, and potential effects on diet-induced thermogenesis and resting energy expenditure. Indirect mechanisms include modulation of the gut microbiota and short-chain fatty acid production, insulin sensitivity, and inflammation. Bidirectional arrows indicate conceptual feedback between mechanisms. Contextual factors, including nut dose, nut type and form, dietary comparator, and individual characteristics, may moderate these pathways. Pathways are presented for conceptual integration and do not indicate equal weighting or confirmed causality. Evidence classification is qualitative and reflects study design, quantity of evidence, and consistency of direction. “Consistent” evidence indicates higher-level human evidence, including systematic reviews and randomized controlled trials, that generally supports the same directional effect, although the magnitude of effect may vary across studies. “Limited” evidence indicates sparse, indirect, primarily mechanistic, or inconsistent evidence that remains less certain. Arrow direction indicates the proposed direction of effect, and line style indicates the certainty of the pathway linking each proximal mechanism to energy balance or body weight outcomes, rather than the relative magnitude of contribution. DIT, diet-induced thermogenesis; REE, resting energy expenditure; SCFA, short-chain fatty acids.

Figure 2 presents a conceptual framework integrating the reviewed mechanisms through which nuts may influence energy balance, while remaining consistent with thermodynamic principles and contemporary dynamic energy balance models. The framework suggests that the expected energy contribution of nuts may be attenuated by direct mechanisms, particularly energy compensation and reduced metabolizable energy. Indirect mechanisms, including effects on gut microbiota, SCFA production, insulin sensitivity, and inflammatory markers, may influence the metabolic environment, supporting long-term energy balance. The framework is explanatory rather than predictive and does not imply that all mechanisms contribute equally.

A key distinction from simplified interpretations of energy balance is the characterization of nuts as complex food matrices rather than primarily calorie-dense foods. Nuts contain unsaturated fats, protein, fiber, vitamins, minerals, and bioactive compounds, including polyphenols such as ellagitannins and proanthocyanidins. The nutritional profile and physical structure may shape their effects on the direct and indirect pathways relevant to energy balance.

The framework incorporates both dose- and time-dependent effects. Higher nut doses provide more calories but also increase exposure to components that may amplify the mechanistic processes involved. This feature of the framework aligns with the evidence from dose–response analyses showing modest downward trends in body weight with higher nut intakes. With respect to time, the framework proposes that much of the caloric contribution of nuts is offset by direct mechanisms in the short term, resulting in weight neutrality. Over longer timeframes, cumulative effects of both direct and indirect mechanisms are proposed to contribute to a modest reduced risk of weight gain. Evidence of potential dose- and time-response relationships was also identified for select mechanisms. For example, subgroup analyses suggest that reductions in some inflammatory markers are more consistently observed at higher nut intakes (≥50 g/day) and with longer study durations (≥12 weeks) (133, 134).

Several limitations are acknowledged. As a structured narrative synthesis rather than a formal systematic review, this review may be subject to selection bias, and not all relevant studies may have been identified. The mechanisms outlined in this framework are not exhaustive, and additional processes may contribute. Evidence strength was classified qualitatively, but no formal certainty grading was conducted. The evidence base also varies across mechanisms. For direct mechanisms, evidence is limited by the number of studies, heterogeneous designs, and short intervention durations, making the long-term impact of these mechanisms on weight regulation unclear. For indirect mechanisms, their contribution to body weight regulation is theoretically supported, but causality has not been established in long-term trials.

The framework also does not quantify the relative importance of contextual factors, including nut type and form, population characteristics, and the dietary context, including whether nuts are substituted for other foods, the types of foods they replace, and the habitual dietary patterns of their consumption. Mechanistic evidence is unevenly distributed across nut types and age groups, with a substantial proportion of studies conducted using almonds and walnuts in adults. This limits the generalizability of the framework across all nut types and ages. Effects may also vary by baseline metabolic health profile, such as insulin sensitivity, body weight status, or broader cardiometabolic risk.

Finally, interpretation of the body weight evidence requires distinction between RCTs and prospective cohort studies. RCTs provide stronger causal evidence and consistently indicate weight neutrality but are generally shorter in duration and may be less able to detect small cumulative effects. Prospective cohort studies provide longer-term evidence but cannot establish causality and are at risk of confounding, with nut consumers consistently reporting healthier overall dietary patterns and lifestyles, and higher socioeconomic status. Nevertheless, the framework integrates evidence from clinical trials, prospective cohorts, and mechanistic studies to provide a biologically plausible interpretation for the observed relationships between nut consumption and body weight.

## 7 Future directions

The proposed framework attempts to provide a mechanistic explanation for why nut consumption does not promote weight gain despite their high calorie density. Future studies should test and refine this framework using longer-term trials that capture dose–response, time-dependent, and contextual effects, including comparisons across nut types and forms, and different dietary comparators. Pre-defined subgroup analyses by age, baseline metabolic health, habitual dietary pattern, eating occasion, and cultural dietary context would help clarify where and for whom the framework is most applicable.

Future mechanistic trials are also required to establish whether the mechanisms causally link increased nut intake to meaningful changes in energy balance. Factorial or mediation-based study designs could help quantify the relative contributions of these pathways and identify whether threshold effects exist. Similar frameworks could also be tested for other healthful but calorie-dense foods, such as extra-virgin olive oil or avocado, to examine whether comparable mechanisms apply.

As the evidence grows, the framework may help to inform a more nuanced translation of research into clinical practice, public health messaging, and food-labeling policy for nuts and other nutrient-rich, calorie-dense foods. Although current evidence does not support interpreting 30 g/day as an upper limit in the context of energy balance, further research is still needed to clarify how different nut intake levels relate to long-term energy balance across different populations and contexts.

## 8 Conclusion

This review proposes an integrated conceptual framework to explain why nut consumption does not typically promote weight gain despite their high calorie density. The framework highlights that nuts may influence energy balance through direct mechanisms, including reduced metabolizable energy and energy compensation, and through indirect mechanisms involving gut microbiota, short-chain fatty acid production, insulin sensitivity, and inflammatory markers.

Although the framework is conceptual and includes pathways for which understanding remains incomplete, it provides an integrated approach to interpret and contextualize existing evidence. Further research is needed to quantify the relative contribution of these pathways and clarify how effects vary by contextual factors including nut type, nut form, dietary context, and population characteristics. Overall, these findings support consideration of the total nutritional composition and physical structure of nuts, whose effects on body weight cannot be inferred from calorie content alone.
