# Supplementary material for: A conceptual framework integrating mechanisms linking nut consumption and energy balance
Source: Front Nutr. 2026 Jul 2;13:1834816. doi: 10.3389/fnut.2026.1834816 (PMC13373753; doi:10.3389/fnut.2026.1834816)
Supplement: Supplementary file 1 [file Table_1.pdf]

Supplementary Material

Supplementary Table S1. Search terms and selection considerations used to inform the structured narrative synthesis

Searches were conducted in PubMed/MEDLINE in May 2025, with no date limits applied. Searches were supplemented by reference list screening.

Research area	Search terms	Selection considerations
Body weight	nut AND (weight OR BMI OR waist circumference) AND systematic review	Prioritized systematic reviews and meta-analyses of RCTs and prospective cohorts assessing body weight and adiposity outcomes.
Satiety / appetite	nut AND (satiety OR hunger OR fullness OR appetite) AND review	Prioritized reviews of RCTs assessing subjective appetite outcomes such as hunger and fullness.
Energy intake / compensation	nut AND energy intake AND review	Prioritized reviews of RCTs examining energy compensation or changes in total dietary intake.
Energy expenditure	nut AND energy expenditure AND systematic review	Prioritized reviews and RCTs assessing diet-induced thermogenesis, resting energy expenditure, postprandial energy expenditure, or substrate oxidation.
Metabolizable energy	nut AND (metabolisable energy OR metabolizable energy) AND systematic review	Prioritized reviews comparing measured metabolizable energy with Atwater-predicted values; mechanistic studies were used to interpret lipid bioaccessibility.
Gut microbiota / SCFA	nut AND gut AND (microbiota OR microbiome) AND review	Prioritized systematic reviews and RCTs assessing gut microbiota composition, SCFA production, or gut-related metabolic outcomes.

Research area	Search terms	Selection considerations
Insulin sensitivity / glycemic control	nut AND (insulin OR glucose) AND systematic review	Prioritized systematic reviews assessing HOMA-IR, fasting insulin, fasting glucose, HbA1c, or related markers.
Inflammation	nut AND (inflammation OR inflammatory) AND systematic review	Prioritized systematic reviews of RCTs assessing inflammatory markers.
Food matrix / nut form	nut AND food AND (form OR structure OR matrix) AND weight	Prioritized mechanistic studies linking nut structure, form, processing, mastication, lipid release, digestion, or metabolizable energy.

Abbreviations: BMI, body mass index; HbA1c, glycated hemoglobin; HOMA-IR, homeostatic model assessment of insulin resistance; RCT, randomized controlled trial; SCFA, short-chain fatty acid.

Note: Study selection prioritized relevance to the conceptual framework, level of evidence, and generalizability. Where newer publications were narrower in scope, including single-nut, population-specific, or non-systematic reviews, broader systematic reviews and meta-analyses were prioritized for general conclusions, with narrower evidence used as supportive context where relevant. Formal exclusion criteria and a PRISMA flow diagram were not applied because this review was not conducted as a formal systematic review.
